# Quinonoids: Therapeutic Potential for Lung Cancer Treatment

**DOI:** 10.1155/2020/2460565

**Published:** 2020-04-06

**Authors:** Hua-Zhong Ying, Chen-Huan Yu, Hao-Kun Chen, Huan-Huan Zhang, Jie Fang, Fang Wu, Wen-Ying Yu

**Affiliations:** ^1^Key Laboratory of Experimental Animal and Safety Evaluation, Hangzhou Medical College, Hangzhou 310013, China; ^2^Key Laboratory of Neuropsychiatric Drug Research of Zhejiang Province, Institute of Materia Medica, Hangzhou Medical College, Hangzhou 310013, China

## Abstract

Lung cancer is the leading cause of cancer-related deaths worldwide. Owing to its high incidence and mortality, the development and discovery of novel anticancer drugs is of great importance. In recent years, many breakthroughs have been achieved in the search for effective anticancer substances from natural products. Many anticancer drugs used clinically and proven to be effective are derived from natural products. Quinonoids, including naphthoquinones, phenanthrenequinones, benzoquinones, and anthraquinones, constitute a large group of natural bioactive compounds that widely exist in higher and lower plant species. Given that most of these compounds possess anticancer effects, they are applied in many cancer studies, especially in lung cancer research. They can promote apoptosis, induce autophagy, and inhibit proliferation, angiogenesis, and cell invasion and migration. Some drugs can enhance anticancer effects when combined with other drugs. Thus, quinonoids have broad application prospects in the treatment of lung cancer. Here, we summarize the previous studies on the antilung cancer activities of quinonoids together with their underlying mechanisms and analyze the common research targets with different effects so as to provide references for the discovery of quinonoids against lung cancer.

## 1. Introduction

Lung cancer is the leading cause of cancer-related deaths worldwide, and the incidence rate showed an increasing trend. The onset of lung cancer is relatively insidious. Most patients are diagnosed late and thus lose the chance to undergo operation. Chemoradiotherapy remains the main treatment method, which can alleviate the illness but has serious side effects [1]. Therefore, searching for safer and more effective antilung cancer drugs is necessary. Natural products are a rich source of discovery and development of antilung cancer drugs. With the emergence and improvement of new technologies, such as genetic techniques, combinatorial chemistry technology, and high-throughput screening for plant secondary metabolite production, safe and effective antilung cancer drugs can be identified and developed from natural sources (including medicinal plants). A large number of preclinical and clinical studies have confirmed that many natural products have potential antilung cancer properties [2]. Thus, developing natural antilung cancer products is important.

Quinonoids are a large group of natural bioactive molecules with unsaturated cyclodiketone structures (quinone structures) [3]. They possess extensive biological activities, such as antidiarrheal, bactericidal, anti-inflammatory, and antiviral activities; inhibit tumor growth and coronary artery expansion; alleviate ultraviolet radiation damage; and improve immune function [3, 4]. Quinonoids serve as vital links in electron transport chains in the metabolic pathway, participating in multiple biological oxidative processes. This redox characteristic accounts for the inherent cytotoxicity of quinonoids. Quinonoids have constituted the largest class of cytotoxins used as anticancer drugs and are frequently used in lung cancer research [5]. Their mechanisms involve a variety of pathways that promote apoptosis, induce autophagy, and inhibit proliferation, angiogenesis, cell invasion, and metastasis. However, the anticancer effects of many quinonoids, especially those against lung cancer, have not been clarified. In the present review, we introduce the distribution of quinonoid plant resources, summarize the antilung cancer activities and underlying mechanisms of quinonoids, and analyze the common targets with different effects to promote the research and development of antilung cancer agents from quinonoids.

## 2. Distribution of Quinonoid Plant Resources

Quinonoids are mainly classified into four types: benzoquinones, naphthoquinones, phenanthrenequinones, and anthraquinones. Among them, anthraquinones and their derivatives are the most common natural active ingredients. Natural quinonoids are widely distributed in more than 100 species of higher and lower plants, many of which are common Chinese herbal medicines. The distribution of common quinonoid plant resources is shown in Table 1.

## 3. Quinonoids in Lung Cancer

In recent years, significant amounts of studies have shown that quinonoids have obvious antilung cancer effect.  Table 2 summarizes some antilung cancer quinonoids and their plant sources. The mechanisms are mainly as follows: inhibition of lung cancer cell growth and proliferation, initiation of apoptosis and autophagy, induction of antiangiogenesis, reduction of invasion and metastasis, and enhancement of chemosensitivity.

### 3.1. Quinonoids Inhibit Proliferation and Induce Apoptosis or Necrosis

Proliferation-related pathways affected by quinonoids mainly include the PI3K/Akt, Ras/MAPK, and JAK/STAT pathways, which are often disordered in cancer progression and thus promote the growth and proliferation of cancer cells. Quinonoids can regulate the cell cycle control system involved in lung cancer progression. This system mainly includes cell cycle protein (cyclin), cyclin-dependent kinases (CDKs), and cyclin-dependent kinase inhibitors (CKIs).

In A549 lung cancer cells, the natural product embelin can inhibit lung cancer through oxidative stress-induced MAPK signaling transduction. The levels of p-p38 and p-JNK are elevated as early as 4 h after embelin administration, resulting in cell apoptosis [6].

Emodin is an anthraquinone derivative from the rhizome of rhubarb and possesses potent anticancer activities. In A549 cells, emodin can induce cell cycle arrest and modify an exogenous apoptotic pathway to inhibit cell growth and initiate apoptosis. Apoptotic characteristics, such as DNA single-strand breakage and DNA fragmentation, are observed in emodin-treated A549 cells. Meanwhile, emodin can upregulate FasL gene expression and downregulate c-Myc cancer gene expression [7]. In addition, emodin can inhibit Anip973 cell proliferation, arrest cell cycle in the G0/G1 stage, decrease the cell proportion at S phase, and trigger the activation of caspase-3, thus inhibiting tumor growth in a dose- and time-dependent pattern [8]. Su et al. [9] found that tribbles homolog 3 (TRIB3) signaling is associated with emodin-induced ER stress-mediated apoptosis in human non-small-cell lung cancer (NSCLC) cell lines. Emodin-induced apoptotic effect decreases by inhibition of ER stress with 4-phenylbutyrate. When TRIB3 is silenced by siRNA, emodin-induced apoptosis is also weakened.

Oxidative damage caused by reactive oxygen species (ROS) plays a crucial role in the emodin-induced apoptosis of mitochondria. Emodin activates the ATM/p53/Bax dependent pathway by inducing ROS generation. The p53 protein increases in A549 cells, which in turn upregulates the level of Bax, and these effects lead to mitochondrial-dependent apoptosis in A549 cells [10]. Additionally, emodin can mediate the production of peroxidase proliferator-activated receptor *γ* (PPAR*γ*) through ERK and adenosine 5′-monophosphate-dependent protein kinase *α* (AMPK*α*), followed by the reduction of pregnancy-specific *β*1-glycoprotein (SP1). This in turn induces insulin-like growth factor binding protein 1 (IGFBP1) gene expression. Thus, synergistic interactions among signal cascade, positive feedback loop, and transcription factors may contribute to the overall responses of emodin on NSCLC cells [11]. Some studies have found that emodin may inhibit NSCLC cell proliferation by downregulating the expressions of excision repair cross-complementary 1 (ERCC1) and DNA repair protein RAD51 homolog 1 (Rad51) with a dose-dependent pattern. The downregulation of these two proteins is associated with the inactivation of the MKK1/2-ERK1/2 signaling pathway [12, 13]. Besides, emodin can inhibit the transcription effect transactivated by the homolog and heterodimer of retinoic acid X receptor *α* (RXR*α*). The exhibition of emodin and its anticancer activity may be associated with the RXR*α* signaling pathway [14].

Plumbagin is a nature naphthoquinone isolated from three families, namely, Droseraceae, Ebenaceae, and Plumbaginaceae, and can inhibit cell proliferation and induce apoptosis in NSCLC cells via NF-*κ*B inactivation. Plumbagin treatment increases the intracellular ROS level; suppresses the activation of NK-*κ*B; inhibits the degradation of I*κ*B*κ*; downregulates the expression of Bcl-2; upregulates the expression of Bak, Bax, and CytC; and promotes the activities of caspase-9 and caspase-3 [15].

*β*,*β*-Dimethylacrylshikonin is one of the active components in the root extracts of *Lithospermum erythrorhizon*. This compound suppresses cell viability in A549 cells at dose- and time-dependent fashion. It can downregulate the expression of the cell inhibitor of apoptosis protein-2 (cIAP-2) and X-linked inhibitor of apoptosis protein (XIAP) and upregulate the levels of Bax and Bak to induce apoptosis of A549 cells. Moreover, *β*,*β*-dimethylacrylshikonin promotes the loss of mitochondrial membrane potential (MMP) and release of CytC in A549 cells; activates caspase-3, caspase-8, and caspase-9; and then cleaves poly ADP-ribose polymerase (PARP) [16].

Chrysophanol is an anthraquinone compound and has been found to induce different types of cancer cell death. It can promote the release of ROS and Ca^2+^ in A549 cells, decrease MMP and adenosine triphosphate level, trigger DNA damage, and stimulate the release of CytC, but it does not activate apoptosis-related proteins, including caspase-8, caspase-3, apoptosis protease-activating factor-1, and apoptosis-inducing factor (AIF). Therefore, the A549 cells treated with this compound show a cellular pattern related to the death of necrotic cells rather than apoptosis in vitro [17].

Tanshinone IIA, tanshinone I, and cryptotanshinone isolated from *Salvia miltiorrhiza* have been reported to exert antilung cancer effects [18]. Tanshinone IIA can inhibit A549 cell proliferation, activate the JNK signal transduction, and trigger caspase cascade apoptosis induced by releasing caspase activators, including CytC [19]. Tanshinone I, which is a possible radiation sensitizer in lung cancer treatment, can significantly inhibit cell proliferation and clone formation so as to promote the radiosensitivity of radioresistant lung cancer cells. Additionally, it can downregulate phosphoribosyl pyrophosphate aminotransferase (PPAT) expression in lung cells, H358-IR and H157-IR. Molecular docking results suggest that tanshinone I is docked well into the binding site of PPAT; therefore, this compound may serve as a novel PPAT inhibitor [20]. Cryptotanshinone can induce immunological antitumor responses; it inhibits proliferation via upregulating p53, downregulating Cdc2 and cyclin B1, and blocking cell cycle at G2/M phase in Lewis lung carcinoma (LLC) cells. Meanwhile, cryptotanshinone promotes mouse and human dendritic cell maturation by upregulating MHC and costimulatory molecules, as well as by the production of TNF-*α*, IL-12 p70, and IL-1*β* [21]. Pretreatment with cryptotanshinone not only can inhibit the basal levels of IGF-1R and Akt phosphorylation but also can block IGF-1-induced phosphorylation of IGF-1R and Akt. This finding indicates that cryptotanshinone suppresses lung cancer cell proliferation and migration by inhibiting the PI3K/Akt pathway mediated by IGF-1R [22].

The STAT3 signal transduction pathway plays a key role in the biology of NSCLC. Yang et al. [23] found that rhein, a lipophilic anthraquinone, might exert its antilung cancer effect by inhibition of STAT3. Rhein can markedly reduce viabilities and induce apoptosis in human lung cancer A549, PC9, and H460 cells. Rhein can upregulate the proapoptotic protein Bax expression and downregulate the antiapoptotic protein Bcl-2 expression. Furthermore, rhein induces G2/M phase arrest in NSCLC cells and inhibits cycle-related protein expression. It was further found in vivo that rhein could significantly suppress H460 tumor growth in xenograft models.

Juglone, a naphthoquinone, isolated from *Juglans regia* is a potential cytotoxic compound against human lung cancer cell lines, and the electron-withdrawing groups at the ortho position of the R moiety of juglone seem to be necessary for the enhancement of its cytotoxic activity [24].

Wan et al. [25] found that aloin may induce A549 cell apoptosis via the ROS-MAPK pathway and p53 phosphorylation. Aloin can disrupt the MMP, elevate cytosolic Ca^2+^ levels, and activate Bak, Bax, p53-upregulated modulator of apoptosis, and phorbol-12-myristate-13-acetate-induced protein 1. It can also promote p53 phosphorylation.

Aloe-emodin can significantly induce apoptosis, release nuclear phosphoric acid from nucleus to cytoplasm, increase the size of a nucleoprotein in the cytoplasm and the number of fragments, and increase activated caspase-3 and PARP shear. It can promote ROS production and activate MAPKs, inactivate the PI3K-Akt pathway, and inhibit cancer cell proliferation [26]. Anoikis induced by aloe-emodin is associated with the expression levels of MAP kinase members [27]. Active oxygen species produced by aloe-emodin in H460 cells can induce DNA single-strand breaks and decrease the mRNA levels of DNA repair enzymes, such as hMTH1, hOGG1, and DNA damage repair gene depurine/apyrimidine endonuclease mRNA [28]. In lung cancer cell H460, photoactivated aloe-emodin can induce anoikis and morphological changes by acting on the cytoskeleton, promote the expression of PKC, and regulate cytoskeleton-related proteins mediated by PKC [29]. ER stress is accompanied by mitochondrial damage in this process. The expression of ER chaperone molecule HSP70, 150 kDa oxygen-regulated protein, and protein disulfide-isomerase increases. ATP synthase is inhibited. Thus, ATP level decreases in a time-dependent manner [30].

Salvicine can induce DNA double-strand breaks and telomere erosion in A549 cells. It can cause telomere binding protein 2 (TRF2) destruction but does not depend on its transcription or proteasome-mediated degradation. On the one hand, TRF2 protects telomeric DNA and genomic DNA. On the other hand, the human anthrax toxin receptor (ATR) and TRF2 regulate each other by inducing DNA damage signal transduction during cancer development. ATR is essential for telomere erosion. Activated ATR increases TRF2 damage induced by salvicine, and reduced TRF2 can enhance ATR function [31]. Salvicine inhibits telomerase activity of human lung adenocarcinoma A549 cells only under high-concentration and short-term irradiation and also suppresses the activity of telomerase more severely than other drugs. In addition, 4 h exposure to salvicine has no effect on the mRNA levels of human telomerase reverse transcriptase (hTERT), human telomerase-associated protein 1 (hTP1), and human telomerase (hTR) in A549 cells [32].

SYUNZ-16 can induce human lung adenocarcinoma cell apoptosis and suppress tumor growth via inhibiting PKB/Akt kinase activity and blocking the Akt/FOXO signaling pathway. Treatment of GLC-82 cells with SYUNZ-16 can decrease Akt phosphorylation and increase annexin V-positive cells and cleaved caspase-3 and PARP fragments. It partially weakens FKHR and FKHRL1 phosphorylation, promotes FKHR accumulation in the nucleus, and upregulates the mRNA levels of Bim and TRADD in tumor cells [33].

Dioscoreanone isolated from *Dioscorea membranacea* Pierre exhibits antiproliferative and cytotoxic activities in A549 cells. It slows down the rate of cell division and arrests cells at the G2/M stage, resulting in an increase in the sub-G1 apoptotic cell population. Additionally, dioscoreanone can increase the values of the Bax/Bcl-2 ratio in drug-treated cells, indicating that dioscoreanone induces apoptosis through the mitochondrial signaling pathway [34].

ASK1 is associated with various diseases induced by oxidative stress. A549 cells treated with denbinobin show increases in the activity of ASK1 and the production of ROS, and denbinobin induces JNK activation and c-Jun phosphorylation. Thus, an AP-1-specific DNA-protein complex forms and the expression of Bim increases. Ultimately, denbinobin leads to A549 apoptosis. During apoptosis, denbinobin can induce MMP loss and promote the release of mitochondrial apoptotic factors. In addition, denbinobin induces the inactivation of Akt, and transfection of A549 cells with wild-type-Akt and constitutively active Akt dramatically suppresses the activation of Bad and cell apoptosis induced by denbinobin [35, 36].

Przewaquinone A isolated from the roots of *Meriandera benghalensis* has strong cytotoxic effect on lung cancer A427 cell lines [37].  Table 3 summarizes the above antilung cancer quinonoids.

### 3.2. Quinonoids Induce Autophagy

Quinonoids can also induce autophagy-related cell death in lung cancer cells (Table 4). The loss of p53 function, through mutations, is involved in the development of various human cancers. Haque et al. [38] found that emodin treatment can upregulate LC3 expression by blocking p53 aggregation and inducing autophagy in A549 cells, which can dissociate the coaggregation of ATG5 and p53 aggregates and restore p53 function.

Plumbagin exhibits potential proautophagic and proapoptotic effects on human NSCLC cells. It can arrest the cell cycle in the G2/M stage and increase the ROS level in both A549 and H23 cells. A crosstalk occurs between autophagy and apoptosis induced by plumbagin. Plumbagin triggers autophagy by inhibiting the PI3K/Akt/mTOR pathway, and the induction of autophagy enhances plumbagin-induced apoptosis [39].

Cryptotanshinone can induce intracellular ROS generation at a dose- and time-dependent pattern, promote LC3 expression, and induce autophagy. However, autophagy induced by cryptotanshinone can be inhibited by N-acetylcysteine (NAC), JNK siRNA, and SP600125. In A549 xenograft nude mice, cryptotanshinone (10 mg·kg^−1^) can dramatically inhibit tumor growth, which can be reversed by cotreatment with NAC. These findings show that cryptotanshinone can induce a prodeath autophagy via the activation of the JNK pathway [40].

### 3.3. Quinonoids Inhibit Angiogenesis and Tumor Metastasis and Invasion

Angiogenesis is a key factor involved in the invasion and metastasis of human lung cancer. Some quinonoids can inhibit angiogenesis and thus prevent lung cancer from progressing.

Emodin can inhibit A549 cell proliferation, migration, and epithelial-mesenchymal transition (EMT) induced by ATP through inhibiting the P2Y receptor-mediated Ca^2+^ increase and NF-*κ*B activation [41]. CXC chemokine receptor 4 (CXCR4) is closely related to the occurrence and development of tumor. Emodin can suppress invasion and migration of A549 cells via downregulating CXCR4 expression; thus, it has the potential to treat metastatic cancers [42].

Recent studies have shown that osteopontin (OPN) can stimulate movement and invasion in A549 cells. However, plumbagin can effectively inhibit OPN-induced ROCK1 expression and the phosphorylation levels of LIM kinase 1/2 and cofilin and reduce OPN-induced FAK and Akt phosphorylation. In vivo studies further demonstrate that plumbagin can suppress lung metastasis [43]. Plumbagin can also suppress the proliferation and invasion of human large-cell lung cancer cell lines by regulating the IL-6/STAT3 pathway [44].

Thymoquinone is an edible redox-active quinone. It can suppress the proliferation, migration, and invasion of A549 cells via the ERK1/2 signaling pathway. The therapeutic potential of thymoquinone as an anti-invasive and antimetastatic drug in human NSCLC treatment has been explored [45].

As indicated by molecular docking analyses, tanshinone IIA can bind well at the VEGFR2 kinase site to form H bond with Cys917 in a unique mode and form *π*-*π* stacking interaction with Val828. This finding indicates that tanshinone IIA can inhibit angiogenesis by targeting VEGF/VEGFR2 protein kinase domains [46].

Three naphthoquinone derivatives (shikonin, acetylshikonin, and isobutyl shikonin) effectively inhibit the migration of HUVECs treated with VEGF. Shikonin has a specific inhibitory effect on the generation of VEGF. These three derivatives can downregulate uPA expression but do not inhibit the expression of its receptor uPAR. Shikonin can also significantly suppress the growth of LLC tumors in vivo, while the effect of its derivatives is relatively mild. The findings show that shikonin and its derivatives have antiangiogenic and antitumorigenic effects [47].

It is well known that IGF-1R activity is closely related to angiogenesis and tumor growth. Tsai et al. [48] found that denbinobin could suppress the growth and microvessel formation of A549 cells by blocking the IGF-1R pathway. It can selectively inhibit IGF-1-mediated proliferation and tube formation of HUVECs but not affect the role of EGF, VEGF, and bFGF. Therefore, denbinobin may be a new type of the IGF-1R kinase inhibitor, which has therapeutic potential for angiogenesis-related diseases, such as lung cancer.

Recent reports suggest that the basic fibroblast growth factor (bFGF) is also associated with angiogenesis in many malignant tumors. Salvicine, which is a naphthoquinone derivative, can effectively inhibit the migration and tube formation of HMECs. Moreover, it can significantly reduce the expression of bFGF mRNA, whereas the expression of VEGF mRNA remains unchanged in A549 cells. Salvicine possibly exerts potential antiangiogenic effect by inhibiting the proliferation, migration, and angiogenesis, which is related to bFGF expression in tumor cells [49]. The effects of quinonoids on the inhibition of angiogenesis, tumor migration, and invasion are summarized in Table 5.

### 3.4. Quinonoids Enhance the Antilung Cancer Effect of Other Drugs

The multidrug resistance (MDR) of lung cancer cells to chemotherapeutic drugs is a major cause in the unsatisfactory effect of chemotherapeutic drugs. Traditional Chinese medicine (TCM) monomers can reverse MDR in lung cancer in many ways and multiple levels, and this feature is a manifestation of the advantages of natural monomers in high-efficiency, low-toxicity, and multitarget antilung cancer treatment [50]. Quinonoids are used in this field. Some quinonoids can attenuate toxicity and increase efficiency and reverse multidrug resistance to lung cancer.

Embelin can enhance TNF-related apoptosis-inducing ligand- (TRAIL-) induced apoptosis in A549 cells. The synergistic antitumor effect of embelin-TRAIL cotreatment might be related to regulation of multiple factors in the TRAIL receptor-mediated apoptotic pathway, such as survivin, Bcl-2, TRAILR2, c-FLIP, and XIAP [51]. High XIAP expression plays an important role in regulating cisplatin resistance in H460 lung cancer cells. Embelin (XIAP inhibitor) or siRNA can increase the activation of caspase-3 by blocking XIAP activity and promote the death of cisplatin-induced H460 cells [52].

Core EMT factors Snail, Slug, and Twist promote cell proliferation, metastasis, and invasion and inhibit the apoptosis of H69AR cells. Compared to doxorubicin- (Dox-) sensitive H69 cells, H69AR can induce Snail, Slug, and Twist expression, whereas emodin combined with Dox can effectively inhibit their expression. The results indicate that emodin can reverse H69AR cell resistance to Dox by inhibiting cell proliferation, cell migration, cell invasion, and EMT [53]. Rad51 plays a critical role in homologous recombination and is highly expressed in cancers tolerant to chemotherapy or radiation. Emodin can enhance mitomycin C- (MMC-) induced cytotoxic effects through reducing the levels of Rad51 and ERK1/2 phosphorylation. By contrast, Rad51 overexpression can protect H1703 or A549 cells from synergistic cytotoxicity mediated by MMC and emodin [54].

Emodin can significantly enhance paclitaxel-elicited apoptosis of A549 cells by upregulating the Bax and active caspase-3 expression and downregulating p-Akt, p-ERK, and Bcl-2 expression. Furthermore, emodin can effectively enhance the anticancer activity of paclitaxel on the A549 xenograft model with no obvious side effect in vivo [55].

Tanshinone IIA can increase TRAIL-mediated apoptosis by upregulating DR5 and downregulating survivin. The processes are induced by selectively activating the PERK/ATF4 signaling pathway and inhibiting STAT3, respectively. These features suggest that tanshinone IIA combined with TRAIL is a novel strategy for the treatment of NSCLC [56]. Tanshinone IIA combined with cyclophosphamide can downregulate Bcl-2, upregulate Bax, suppress neovascularization of lung cancer, and boost the immune functions [57]. Tanshinone IIA and Dox can synergistically induce apoptosis, inhibit migration of A549 cells, and block cell cycle in the S and G2 stages. Compared with the single drug use, a combined treatment can further downregulate the protein levels of VEGFR2, VEGF, caspase-3, p-PI3K, p-Akt, and Bcl-2. The results of molecular docking show that tanshinone IIA can be bound into the active pockets of all the proteins tested compared with Dox [58]. Tanshinone IIA combined with cisplatin exerts a synergistic inhibitory effect on A549 and PC9 cells. A combination treatment induces apoptosis, impairs invasion and migration, and blocks the cell cycle at the S stage in NSCLC cells. Molecular docking and KEGG analysis show that tanshinone IIA may mainly affect the PI3K/Akt pathway [59].

The combination of rhein lysinate and paclitaxel can significantly inhibit H460 and A549 cell proliferation. Rhein lysinate enhances paclitaxel-elicited cytotoxicity via downregulating the activity of ERK and upregulating the expression of cleaved PARP and caspase-3 [60].

The expressions of endogenous Nrf2 and its target genes, including HO-1, MRP1, GCLC, GCLM, and NQO1, are much higher in cisplatin-resistant A549 cells than in A549 cells. Cryptotanshinone combined with cisplatin can make cisplatin-resistant cells sensitive to cisplatin, leading to cell death and apoptosis, whereas the reversal effect can be abolished by knockdown of Nrf2. Notably, cryptotanshinone can significantly diminish the expression of Nrf2, thus reducing the expression levels of Nrf2 target genes. Therefore, cryptotanshinone may develop into a novel anticancer chemotherapy sensitizer by inhibiting the Nrf2 pathway [61]. Cryptotanshinone can promote TRAIL-mediated apoptosis by upregulating DR5, which can convert TRAIL-resistant A549 cells into sensitive ones. The knockdown of DR5 abolishes the enhanced effect of cryptotanshinone on TRAIL response. The induction of DR5 by cryptotanshinone is dependent on CCAAT/CHOP, not p53.

The CHOP knockdown abolishes cryptotanshinone-mediated DR5 expression and the enhancement of TRAIL-induced cell death [62]. The effects of quinonoids in enhancing the antilung cancer effects of other drugs are summarized in Table 6.

## 4. Discussion

Different quinonoids with similar structures belong to a large category, but they have different anticancer effects. Targets involve the multiple pathways of cancer progression, with different emphases on inducing lung cancer cell apoptosis, suppressing proliferation and metastasis, and enhancing the efficacy of anticancer drugs. Thus, quinonoids have wide application prospects. Recently, plentiful research focused on anti-inflammatory, antibacterial, and antioxidative effects of quinonoids. However, with the discovery of their antitumor effects, researchers have started to pay considerable attention to the close relationship between the two effects. Inflammatory stimulation can induce and promote cancers. Intervention in the inflammatory microenvironment of tumors can reshape immunity, enhance the killing of tumor cells, and inhibit tumors. For example, emodin can regulate a series of inflammatory cytokines, such as TNF-*α*, thereby reversing the immunosuppressive rates of tumor microenvironments and promoting tumor cell apoptosis [63]. The continuous activation of the NF-*κ*B pathway can increase antiapoptotic gene Bcl-2 expression, upregulate expression of proliferation-related proteins CDK1 and CDK2, and promote cell cycle, thus promoting cancer cell proliferation. Additionally, the process can lead to the upregulation of MMP expression and degradation of the extracellular matrix, thus facilitating the local infiltration of cancer cells [64–66]. The NF-*κ*B pathway activation can also upregulate angiogenesis-related factor expression, such as VEGF, and promote the metastasis and invasion of cancer [67, 68]. The Nrf2 pathway can generate detoxification and antioxidant effects [69]. The regulation of these pathways by quinonoids may be the main mechanism of their antilung cancer effects. Previous studies have demonstrated that embelin, rhein, and emodin can suppress the activity of the NF-*κ*B-mediated inflammatory pathway, and cryptotanshinone can play an anticancer role by activating the Nrf2 pathway.

Most of the TCMs derived from these quinonoids have purgative and anticoagulant effects. Particularly, anthraquinones are widely distributed in purgative drugs. For example, *Rheum palmarum*, *Aloe vera*, *Folium Sennae*, and *Polygonum bistorta* contain a large number of anthraquinones. These drugs may regulate lung function by acting on the intestinal microenvironment.

Quinonoids are extensively distributed in the plant kingdom and possess a wide spectrum of pharmacological properties. However, recent studies on safety of quinonoids have found that some quinonoids can cause toxicity to the digestive system, urinary system, reproductive system, and immune system; the degree of toxicity mainly depends on dose and exposure time [70–73]. Many reports showed that aloe-emodin exerted hepatotoxicity and nephrotoxicity by inducing apoptosis in the Fas death, mitochondrial, and ER stress-triggered signaling pathway [70, 71]. In addition, emodin could also lead to reproductive toxicity, particularly in high doses and/or long-term use. In male reproductive organs/tissues, the toxicity study of emodin indicated that it has testicular toxicity because of the disruption of the expression of testicular genes. Therefore, long-term administration of high doses of anthraquinone extracts should be avoided altogether during pregnancy [72]. The investigations of potential genotoxicity of plumbagin demonstrated that it can induce significant DNA damage in a dose-dependent manner [74], while the evaluation of cytotoxic properties of plumbagin on SKMEL 28 melanoma cell lines and human lymphocytes showed that a very low concentration of plumbagin is sufficient to induce cytotoxic effect on normal cells [73].

Furthermore, some quinonoids are also known to have poor intestinal absorption, short elimination half-life, and low bioavailability in vivo [75], which limit their clinical application further. Traditional drug delivery systems would not be the best method of application of these quinonoids. Therefore, further research studies are needed to improve the bioavailability of quinonoids, reduce toxicity, and enhance their efficacy. Zhou et al. [76] developed a novel hydrogel-based drug delivery system for the delivery of rhein. This rhein-chitosan hydrogel exhibited superior characteristics including sustained release and low toxicity. Aithal et al. [77] compared the pharmacokinetic, biodistribution, pharmacodynamic, and toxicity profiles of free juglone with a sterically stabilized liposomal form. The results showed that sterically stabilized liposomes (SSLs) of juglone exhibited enhanced anticancer activity in vitro and in vivo with reduced toxicity profiles. SSLs also significantly improved the pharmacokinetic and biodistribution attributes of juglone resulting in enhanced plasma half-life and higher tumor accumulation in vivo. Zhang et al. [78] investigated the pharmacokinetic characteristics of rhubarb anthraquinones in rats after being orally administered with rhubarb and rhubarb total free anthraquinone oral colon-specific drug delivery granules. The results found that oral colon-specific drug delivery technology made anthraquinone aglycone to colon-specific release after oral administration. This allowed anthraquinones to not only play the corresponding purgative effect but also avoid intestinal absorption and promote excretion and thereby greatly reduced the nephrotoxicity of anthraquinones. Thus, further development of quinonoids can be focused on modification of pharmaceutical formulation to improve pharmacokinetic properties. In addition, a dose-ranging study in animal models should be performed to obtain information on the safety window of effective and toxic doses for extrapolation to humans.

## Figures and Tables

**Table 1:** Distribution of common quinonoid plant resources [3, 4].

Type	No.	Latin name	Families	Quinonoids in plants
Benzoquinones	1	*Embelia laeta*	Myrsinaceae	Embelin
	2	*Ardisia oxyphylla*	Myrsinaceae	Embelin, rapanone
	3	*Ardisia crenata*	Primulaceae	Rapanone
	4	*Ailanthus altissima*	Simaroubaceae	2,6-Dimethoxy-p-benzoquinone
	5	*Dalbergia odorifera*	Papilionaceae	Claussequinone
	6	*Acanthopanax senticosus*	Araliaceae	2,6-Dimethoxybenzoquinone

Naphthoquinones	1	*Lithospermum erythrorhizon*	Boraginaceae	*β*,*β*-Dimethylacrylshikonin, deoxyshikonin, acetylshikonin, L-shikonin
	2	*Catalpa*	Bignoniaceae	Lapachol
	3	*Plumbago zeylanica*	Plumbaginaceae	Plumbagin
	4	*Juglans regia*	Juglandaceae	Juglone
	5	*Medicago sativa*	Leguminosae	Vitamin K_1_
	6	*Salvia prionitis* Hance	Labiatae	Saprorthoquinone, sapriparaquinone, salvicine

Phenanthrenequinones	1	*Salvia miltiorrhiza*	Labiatae	Nordanshenquinone, cryptosalvianoquinone, tanshinones I and IIA
	2	*Salvia aerea*	Labiatae	Przewaquinone A-F
	3	*Salvia trijuga*	Labiatae	Trijuganones A and B, tanshinones I and IIA
	4	*Rosmarinus officinalis*	Labiatae	Cryptosalvianoquinone, horminone, taxodione
	5	*Asparagus cochinchinensis*	Asparagaceae	Dioscoreanone
	6	*Dendrobium nobile*	Orchidaceae	Denbinobin

Anthraquinones	1	*Rheum palmarum*	Polygonaceae	Emodin, physcion, and rhein
	2	*Fallopia multiflora*	Polygonaceae	Emodin, physcion, and rhein
	3	*Reynoutria japonica*	Polygonaceae	Fallacinolw-O-acetate, emodin
	4	*Morinda officinalis*	Rubiaceae	Physcion, rubiadin
	5	*Rubia cordifolia*	Rubiaceae	Munjistindimethylether
	6	*Arisaema erubescens*	Rubiaceae	Digitoquinone
	7	*Folium Sennae*	Leguminosae	Emodin and sennosides A, B, C, and D
	8	*Semen cassiae*	Leguminosae	Emodin, chrysophanol, rhein, and physcion
	9	*Astragalus propinquus* Schischkin	Leguminosae	Aloe-emodin, rhein, emodin, chrysophanol, and physcion
	10	*Polygonatum sibiricum*	Liliaceae	Polygonaquinone
	11	*Ophiopogon japonicus*	Liliaceae	Emodin and chrysophanol
	12	*Aloe vera*	Asphodelaceae	Aloe-emodin and aloin
	13	*Tripterygium wilfordii*	Celastraceae	1,8-Dihydroxy-4-hydroxymethyl anthraquinone
	14	*Curcuma longa*	Zingiberaceae	2-Hydroxymethylhydrazine
	15	*Sargentodoxa cuneata*	Lardizabalaceae	Chrysophanol

**Table 2:** Common antilung cancer quinonoids.

No.	Compounds	Type	Plant sources
1	Embelin	Benzoquinones	*Embelia laeta*, *Ardisia oxyphylla*
2	Thymoquinone	Benzoquinones	*Nigella damascena*
3	Plumbagin	Naphthoquinones	*Plumbago zeylanica*
4	*β*,*β*-Dimethylacryloylshikonin	Naphthoquinones	*Lithospermum erythrorhizon*
5	Juglone	Naphthoquinones	*Juglans regia*
6	Salvicine	Naphthoquinones	*Salvia prionitis* Hance
7	Acetylshikonin	Naphthoquinones	*Lithospermum erythrorhizon*
8	Alkannin (SYUNZ-16)	Naphthoquinones	*Lithospermum erythrorhizon*
9	Emodin	Anthraquinones	*Rheum palmarum*, *Fallopia multiflora*, *Cassia senna*
10	Chrysophanol	Anthraquinones	*Rheum palmarum*, *Sargentodoxa cuneata*
11	Rhein	Anthraquinones	*Rheum palmarum*, *Fallopia multiflora*
12	Aloin	Anthraquinones	*Aloe vera*
13	Aloe-emodin	Anthraquinones	*Aloe vera*, *Semen cassiae*
14	Tanshinone IIA	Phenanthrenequinones	*Salvia miltiorrhiza*, *Salvia trijuga*
15	Cryptotanshinone	Phenanthrenequinones	*Salvia miltiorrhiza*, *Rosmarinus officinalis*
16	Dioscoreanone	Phenanthrenequinones	*Asparagus cochinchinensis*
17	Denbinobin	Phenanthrenequinones	*Dendrobium nobile*
18	Przewalanshinguinone A	Phenanthrenequinones	*Salvia aerea*

**Table 3:** Effect of quinonoids on inhibiting proliferation and inducing apoptosis or necrosis of lung cancer.

Quinonoids	Cell lines or animal models	Doses	Targets	Pharmacodynamic effects	Ref.
Embelin	A549 cells	2.5~25 *μ*M	p38, JNK, ERK1/2	Induce apoptosis	[6]

Emodin	A549 cells	18.5~129.5 *μ*M	FasL, c-Myc	Inhibit cell growth and induce apoptosis	[7]
	Anip973 cells	10~60 *μ*M	Caspase-3	Induce apoptosis, arrest cell cycle	[8]
	A549 and H1299 cells A549 xenograft nude mice	20~80 *μ*M 50 mg/kg	TRIB3, NF-*κ*B	Induce apoptosis, suppress tumor growth	[9]
	A549 cells	50 *μ*M	p53, Bax, CytC, caspase-3	Induce apoptosis	[10]
	A549 and H1975 cells A549 xenograft nude mice	50 *μ*M 25~50 mg/kg	AMPK*α*, ERK1/2, PPAR*γ*, IGFBP1, Sp1	Arrest cell cycle, inhibit tumor growth	[11]
	A549 and SK-MES-1 cells	70 and 40 *μ*M	ERCC1, Rad51	Inhibit proliferation	[12]
	H1650, A549, H520, and H1703 cells	10~100 *μ*M	ERCC1, Rad51, MKK1/2, ERK1/2	Induce cytotoxicity	[13]
	H460 cells	1~50 *μ*M	RXR	Inhibit proliferation, induce apoptosis	[14]

Plumbagin	A549, H292, H460 cells	3~18 *μ*M	NK-*κ*B, caspase-9, caspase-3, Bcl-2, Bax, Bak, CytC, I*κ*B	Inhibit proliferation, induce apoptosis	[15]

*β*,*β*-Dimethylacrylshikonin	A549 cells	7.5~15 *μ*M	p38, caspase-9, caspase-8, caspase-3, PARP, CytC	Inhibit proliferation, induce apoptosis	[16]

Chrysophanol	A549 cells	1~100 *μ*M	CytC	Inhibit proliferation, induce necrosis	[17]

Tanshinone IIA	H1299, A549, SPCA-1, HCC827, and BEAS-2B cells	2~4 *μ*M	miR-32, AURKA	Inhibit proliferation, promote apoptosis, and block cell cycle	[18]
	A549 cells	2.5~40 *μ*M	JNK, caspase-9, caspase-3, Bax, CytC	Induce apoptosis	[19]

Tanshinone I	H358-IR and H157-IR cells	10 *μ*M	PPAT	Inhibit proliferation and clone formation	[20]

Cryptotanshinone	LLC cells	1.1~67.5 *μ*M	p53, cyclin B1, and Cdc2	Inhibit proliferation	[21]
	A549 and H1299 cells	1~50 *μ*M	IGF-1R, Akt	Inhibit IGF-1-induced proliferation	[22]

Rhein	PC9, H460, and A549 cells H460 xenograft nude mice	30~100 *μ*M 60~100 mg/kg	p-STAT3, Bcl-2, Bax, MDM2, Cdc2, p53, and cyclin B1	Induce apoptosis, block cell cycle, and suppress tumor growth	[23]

Juglone	NCI-H322 and A549 cells	2~50 *μ*M	—	Induce cytotoxicity	[24]

Aloin	A549 cells	100~400 *μ*M	p53, c-Jun/p38, Bcl-2	Inhibit cell proliferation and induce apoptosis	[25]

Aloe-emodin	H520, H226, SK-MES-1, and H1299 cells H520 xenograft nude mice	2.5~40 *μ*M 20 mg/kg	Caspase-3, PARP, caspase-8, caspase-9, MAPKs, PI3K/Akt	Inhibit cell proliferation, promote apoptosis, and suppress tumor growth	[26]
	H460 cells	10~40 *μ*M	Caspase-3, caspase-7, caspase-8, and caspase-9, MAPK, *α*-actinin, HSP27, p38, Bax/Bcl-2	Induce anoikis	[27]
	H460 cells	40 *μ*M	hMTH1, hOGG1, APE	Induce DNA damage and apoptosis	[28]
	H460 cells	20 *μ*M	PKC, RHO, p38, HSP27, FAK	Induce cell death	[29]
	H460 cells	40 *μ*M	HSP60, HSP70	Induce cell death	[30]

Salvicine	A549 cells	25~100 *μ*M	TRF2, *γ*-H2AX, ATM, ATR, p-p53, Hif1*α*	Initiate telomere erosion and DNA double-strand breaks	[31]
	A549 cells	6.25~50 *μ*M	hTERT, hTP1, hTR	Inhibit the telomerase activity	[32]

Alkannin	GLC-82	0.18~21.8 *μ*M	Caspase-3, PARP, p-GSK3*α*/*β*, p-Akt, p-FKHRL1, TRADD, Bim	Inhibit cell proliferation and induce apoptosis	[33]

Dioscoreanone	A549 cells	0.35~35 *μ*M	Bax/Bcl-2, caspase-3	Induce apoptosis	[34]

Denbinobin	A549 cells	20 *μ*M	ASK1, JNK, AP-1, c-Jun, Bim	Induce apoptosis	[35]
	A549 cells	1~20 *μ*M	CytC, Smac, AIF, Bad, Bcl-xL, Akt	Induce apoptosis	[36]

Przewaquinone A	A427 cells	—	—	Induce cytotoxicity	[37]

**Table 4:** Effect of quinonoids in inducing lung cancer autophagy.

Quinonoids	Cell lines or animal models	Doses	Targets	Pharmacodynamic effects	Ref.
Emodin	A549 cells	10~20 *μ*M	ATG5, p53, LC3	Induce autophagic cell death	[38]
Plumbagin	A549 cells, H23 cells	0.5~10 *μ*M	PI3K, Akt, p38, mTOR, LC3I, LC3II, Beclin1	Induce apoptotic and autophagic cell death and block cell cycle	[39]
Cryptotanshinone	A549 cells, A549 xenograft nude mice	1.25~40 *μ*M 10 mg/kg	LC3I, p-JNK, LC3II	Induce autophagic cell death and inhibit tumor growth	[40]

**Table 5:** Effect of quinonoids in inhibiting angiogenesis, tumor migration, and invasion.

Quinonoids	Cell lines or animal models	Doses	Targets	Pharmacodynamic effects	Ref.
Emodin	A549 cells	0.1~10 *μ*M	P2Y receptors, NF-*κ*B	Inhibit cell proliferation, migration, and EMT	[41]
	A549 cells	50~100 *μ*M	CXCR4, CXCL12, HER2, NF-*κ*B	Inhibit cell migration and invasion	[42]

Plumbagin	A549 cells A549 xenograft nude mice	1~8 *μ*M 5 mg/kg	FAK, Akt, ROCK1, LIMK1/2, cofilin	Reduce OPN-induced invasion, suppress lung metastasis	[43]
	L9981 and NL9980 cells	3~12 *μ*M	IL-6, STAT3	Inhibit cell proliferation and invasion	[44]

Thymoquinone	A549 cells	5~160 *μ*M	ERK1/2, PCNA, cyclin D1, MMP2, and MMP9	Inhibit cell proliferation, migration, and invasion	[45]

Tanshinone IIA	A549 cells	2.5~80 *μ*M	VEGF, VEGFR2	Inhibit proliferation by blocking cell cycle and inducing apoptosis and inhibit angiogenesis	[46]

Acetylshikonin	A549 cells LLC-bearing mice	5~20 *μ*M 2 mg/kg	uPA	Inhibit angiogenesis and tumorigenesis	[47]

Denbinobin	A549 cells A549 xenograft mice	0.1~10 *μ*M 25 mg/kg	IGF-1R, ERK, Akt, mTOR, p70S6K, 4EBP, and cyclin D1	Inhibit IGF-1-induced angiogenesis in vitro and in vivo	[48]

Salvicine	A549 cells	0.625~200 *μ*M	bFGF	Inhibit angiogenesis	[49]

**Table 6:** Effect of quinonoids in enhancing antilung cancer effect of other drugs.

Quinonoids	Cell lines or animal models	Doses	Targets	Pharmacodynamic effects	Ref.
Embelin	A549 cells	6.25~100 *μ*M	TRAILR2, XIAP, survivin, Bcl-2, c-FLIP	Enhance TRAIL-induced apoptosis	[51]
	H460 cells	25 *μ*M	XIAP, caspase-3	Enhance cisplatin-induced apoptosis	[52]

Emodin	H69AR cells	1~50 *μ*M	Twist, Snail, Slug, NF-*κ*B	Reverse the resistance of H69AR cells to Dox	[53]
	H1703 and A549 cells	60~200 *μ*M	Rad51, ERK1/2	Enhance MMC-induced cytotoxicity	[54]
	A549 cells A549 xenograft nude mice	5~40 *μ*M 50 mg/kg	Bax, Bcl-2, caspase-3, p-Akt, and ERK	Enhance antitumor effect of paclitaxel	[55]

Tanshinone IIA	A549, H596, H1299, Calu-1, and H460 cells	1~60 *μ*M	TRAIL, DR5, STAT3, PERK/ATF4, survivin	Enhance TRAIL-induced apoptosis	[56]
	LLC-bearing mice	15 mg/kg	Bax, Bcl-2	Combine with cyclophosphamide to inhibit neovascularization and enhance immune function	[57]
	A549 and PC9 cells	5~80 *μ*M	Cleaved caspase-3, Bax, VEGF, VEGFR2, p-PI3K, p-Akt, Bcl-2	Improve the sensitivity of Dox to NSCLC cells	[58]
	A549, PC9, H1299, and SPA-A1 cells A549 xenograft nude mice	1.25~40 *μ*M 7.5~15 mg/kg	Bax, cleaved caspase-3, Bcl-2, caspase-3, p-Akt, and p-PI3K	Combination with cisplatin synergistically inhibits NSCLC in vitro and in vivo	[59]

Rhein	H460 and A549 cells	100 *μ*M	Bcl-2, NF-*κ*B, PARP, ERK, caspase-3	Enhance the inhibitory effect of paclitaxel on cell proliferation	[60]

Cryptotanshinone	A549 and A549/DDP cells	2.5~10 *μ*M	Nrf2, GCLC, GCLM, HO-1, NQO1, MRP1, MAPKs, Akt, STAT3	Restore the sensibility of A549/DDP cells towards cisplatin	[61]
	A549 cells	20 *μ*M	CHOP, DR5	Restore TRAIL sensitivity in TRAIL-resistant cancer cells	[62]
